# Supplementary material for: The Lack of Synergy between Carvedilol and the Preventive Effect of Dexrazoxane in the Model of Chronic Anthracycline-Induced Cardiomyopathy
Source: Int J Mol Sci. 2023 Jun 15;24(12):10202. doi: 10.3390/ijms241210202 (PMC10298964; doi:10.3390/ijms241210202)
Supplement: Supplementary file 1 [file ijms-24-10202-s001.zip › ijms-2425470-supplementary.pdf]

Table S1. Echocardiogram parameter statistical data. Data are presented as *p* value ($n = 10$). Significance: *$p \le 0.05$; **$p \le 0.01$, ***$p \le 0.001$. The analysis was conducted using one-way ANOVA with Tukey's post-hoc test.

Parameter	Group	*p* value *vs.* CTR[a]		*p* value *vs.* DOX[a]		*p* value *vs.* DOX+DEX+CVD[a]	
Ejection Fraction	DOX_I	ns	0.77	-	-	-	-
	DOX_{II}	*	0.0186	-	-	-	-
	DOX + DEX + CVD_I	ns	0.2685	ns	0.9998	-	-
	DOX + DEX + CVD_{II}	***	<0.0001	ns	0.994	-	-
	DOX + DEX_I	ns	0.2278	ns	0.9994	ns	>0.9999
	DOX + DEX_{II}	ns	0.85	ns	0.6713	*	0.0468
	DOX + CVD_I	***	<0.0001	*	0.0218	ns	0.1109
	DOX + CVD_{II}	***	0.0001	ns	0.9692	ns	>0.9999
Fractional Shortening	DOX_I	ns	0.9676	-	-	-	-
	DOX_{II}	ns	0.1207	-	-	-	-
	DOX + DEX + CVD_I	ns	0.2384	ns	0.963	-	-
	DOX + DEX + CVD_{II}	***	0.0002	ns	0.7752	-	-
	DOX + DEX_I	ns	0.1332	ns	0.8877	ns	>0.9999
	DOX + DEX_{II}	ns	0.7752	ns	0.9893	ns	0.1207
	DOX + CVD_I	***	0.0003	*	0.0403	ns	0.6541
	DOX + CVD_{II}	ns	0.1103	ns	>0.9999	ns	0.8611
Left ventricular end-diastolic diameter	DOX_I	ns	0.5012	-	-	-	-
	DOX_{II}	ns	>0.9999	-	-	-	-
	DOX + DEX + CVD_I	ns	0.9142	ns	>0.9999	-	-
	DOX + DEX + CVD_{II}	ns	>0.9999	ns	0.9998	-	-
	DOX + DEX_I	ns	0.9989	ns	>0.9999	ns	>0.9999
	DOX + DEX_{II}	ns	>0.9999	ns	0.9557	ns	>0.9999
	DOX + CVD_I	ns	0.9927	ns	0.998	ns	>0.9999
	DOX + CVD_{II}	ns	>0.9999	ns	0.9877	ns	>0.9999
Left atrial diameter	DOX_I	ns	0.6472	-	-	-	-
	DOX_{II}	ns	0.3945	-	-	-	-
	DOX + DEX + CVD_I	ns	0.9564	ns	0.9999	-	-
	DOX + DEX + CVD_{II}	ns	>0.9999	ns	0.3314	-	-
	DOX + DEX_I	ns	>0.9999	ns	0.6123	ns	0.9485
	DOX + DEX_{II}	ns	0.3418	ns	>0.9999	ns	0.2711
	DOX + CVD_I	ns	>0.9999	ns	0.6931	ns	0.9718
	DOX + CVD_{II}	**	0.007	ns	0.9874	**	0.0047
Ascending aorta diameter	DOX_I	ns	>0.9999	-	-	-	-
	DOX_{II}	ns	>0.9999	-	-	-	-
	DOX + DEX + CVD_I	ns	>0.9999	ns	0.9989	-	-
	DOX + DEX + CVD_{II}	ns	0.946	ns	0.5662	-	-

	DOX + DEX_I	ns	>0.9999	ns	>0.9999	ns	0.9999
	DOX + DEX_{II}	ns	0.9889	ns	0.9174	ns	>0.9999
	DOX + CVD_I	ns	0.8133	ns	0.9694	ns	0.5516
	DOX + CVD_{II}	*	0.0166	*	0.0209	ns	0.4209

Abbreviations: CVD, carvedilol; DEX, dexrazoxane; DOX, doxorubicin; ns, not statistically significant
[a] With relation to a particular time frame
